# Whole-Genome Sequencing and Fine Map Analysis of *Pholiota nameko*

**DOI:** 10.3390/jof11020112

**Published:** 2025-02-03

**Authors:** Yan He, Bo Liu, Xiaoqi Ouyang, Mianyu He, Hongyan Hui, Bimei Tang, Liaoliao Feng, Min Ren, Guoliang Chen, Guangping Liu, Xiaolong He

**Affiliations:** 1 College of Life Sciences, Yan’an University, Yan’an 716000, China; 13165776334@163.com (Y.H.); liubo4552@126.com (B.L.); whx1925@outlook.com (X.O.); 19991455927@163.com (M.H.); hhy554999333@126.com (H.H.); t2679758828@163.com (B.T.); fengliaoliao@yau.edu.cn (L.F.); minren_edu@163.com (M.R.); gcl@yau.edu.cn (G.C.); 2 Research and Development Centre of Ecological and Sustainable Application of Microbial Industry of the Loess Plateau in Shaanxi Province, Yan’an University, Yan’an 716000, China

**Keywords:** *Pholiota nameko*, genome, edible mushroom, mating locus, CAZymes, pan-genomics

## Abstract

*Pholiota nameko* (T. Ito) S. Ito and S. Imai is an emerging wild mushroom species belonging to the genus *Pholiota*. Its unique brown–yellow appearance and significant biological activity have garnered increasing attention in recent years. However, there is a relative lack of research on the biological characteristics and genetics of *P. nameko*, which greatly limits the potential for an in-depth exploration of this mushroom in the research fields of molecular breeding and evolutionary biology. This study aimed to address that gap by employing Illumina and Nanopore sequencing technologies to perform whole-genome sequencing, de novo assembly, and annotation analysis of the *P. nameko* ZZ1 strain. Utilizing bioinformatics methods, we conducted a comprehensive analysis of the genomic characteristics of this strain and successfully identified candidate genes associated with its mating type, carbohydrate-active enzymes, virulence factors, pan-genome, and drug resistance functions. The genome of *P. nameko* ZZ1 is 24.58 Mb in size and comprises 33 contigs, with a contig N50 of 2.11 Mb. A hylogenetic analysis further elucidated the genetic relationship between *P. nameko* and other *Pholiota*, revealing a high degree of collinearity between *P. nameko* and ZZ1. In our enzyme analysis, we identified 246 enzymes in the ZZ1 genome, including 68 key carbohydrate-active enzymes (CAZymes), and predicted the presence of 11 laccases, highlighting the strain’s strong potential for cellulose degradation. We conducted a pan-genomic analysis of five closely related strains of *Pholiota*, yielding extensive genomic information. Among these, there were 2608 core genes, accounting for 21.35% of the total genes, and 135 dispensable genes, highlighting significant genetic diversity among *Pholiota* and further confirming the value of pan-genomic analysis in uncovering species diversity. Notably, while we successfully identified the *A*-mating-type locus, composed of the homeodomain protein genes HD1 and HD2 in ZZ1, we were unable to obtain the *B*-mating-type locus due to technical limitations, preventing us from acquiring the pheromone receptor of the *B*-mating-type. We plan to supplement these data in future studies and explore the potential impact of the *B*-mating-type locus on the current findings. In summary, the genome data of ZZ1 presented in this study are not only valuable resources for understanding the genetic basis of this species, but also serve as a crucial foundation for subsequent genome-assisted breeding, research into cultivation technology, and the exploration of its nutritional and potential medicinal value.

## 1. Introduction

*Pholiota nameko* (T. Ito) S. Ito and Imai (Strophariaceae), commonly called the “*Nameko*” mushroom, is characterized by its bright appearance, smooth and crisp texture, and delicious taste. It is a highly regarded low-calorie, low-fat edible mushroom [1,2]. As one of the top five artificially cultivated edible fungi globally, *P. nameko* is naturally distributed across the United States, Japan, South Korea, China, and Europe [3]. Since the mid-1970s, mushroom cultivation has expanded from southern Liaoning Province in China to northern regions such as Hebei, Liaoning, and Heilongjiang. Notably, Liaoning Province accounts for 32.32% of the country’s total mushroom production [4]. In addition to their tender texture and delightful taste, these mushrooms are rich in various nutrients, including protein, amino acids, polysaccharides, calcium, iron, sodium, and vitamin B12 [5]. *P. nameko* has been shown to have multiple health benefits, including enhancing immune function, cognitive function, vision, and endurance [6]. Further research indicates that the active compounds in *P. nameko* have pharmacological effects, such as antioxidant activity, immune enhancement, blood lipid reduction, and blood coagulation lowering, and antithrombotic effects [7]. Additionally, the viscous substance secreted on the surface of the mushroom cap contains nucleic acids, exhibits tumor inhibitory effects and positively influences both brain health and physical fitness [2,3,4]. A comprehensive evaluation of the protein nutritional value index of *P. nameko* mycelium and fruiting bodies revealed that the mycelium outperforms the fruiting bodies in the amino acid score, chemical score, essential amino acid index, biological value, nutritional index, and amino acid ratio coefficient scores [8]. However, the fruiting bodies have greater nutritional value and have thus become increasingly popular among consumers in recent years.

Sawdust and cottonseed hulls are primarily used as the growth substrates when cultivating the wood-decaying fungus *P. nameko.* Its cultivation has evolved from using partially decomposed to fully decomposed materials, and from tray to bag cultivation [9]. Genetically, *P. nameko* exhibits a typical bipolar heterothallic mating-type system, possessing only one mating-type locus within its mycelium, yet containing two mating-type loci [10]. Although the bioactive compounds and mating system of *P. nameko* have been extensively studied, molecular and genetic research remains relatively limited due to the scarcity of in-depth genomic information [11].

In recent years, the emergence of high-throughput sequencing and single-molecule real-time sequencing technologies has significantly improved the assembly continuity of mushroom genomes [12]. These advancements have led to significant improvements over Illumina sequencing, resulting in substantial progress in genome sequencing for species such as *Lentinula edodes* [13], *Agaricus bitorquis* [14], *Stropharia rugosoannulata* [15], *Gomphus purpuraceus* [16], and *Volvariella volvacea*, which has greatly facilitated the artificial cultivation and strain selection of these species [17].

The genus *Pholiota* encompasses an array of species with both medical and culinary significance, including *Pholiota squarrosa*, *Pholiota aurivella*, and *Pholiota adiposa*; these species are diverse but exhibit only minor morphological differences [18]. While *P. nameko* has been extensively studied in production practices, our understanding of its molecular mechanisms remains inadequate [19]. Additionally, in this article, we discuss related topics such as genomic repetitive sequences, carbohydrate-active enzymes (CAZymes), virulence factors, and drug resistance mechanisms [20]. Our analysis of the *P. nameko* genome sequence significantly contributes to elucidating the molecular mechanisms and evolutionary history of this important edible fungus, as well as notably advances the current understanding of the genus *Pholiota* [21].

## 2. Materials and Methods

### 2.1. Strains and Culture Conditions of the Samples

The mushroom strain “Zaozhuang No. 1” (ZZ1) was kindly provided by the edible fungi research team at Yan’an University, Shaanxi Province, China, which maintains the strain for its long-term preservation. To ensure accurate strain identification, we employed a method that combined hyphal morphology observation with internal transcribed spacer (ITS) sequence analysis. This approach unambiguously confirmed that the sample was *P. nameko* [22].

To obtain an active mycelium for subsequent experiments, the ZZ1 master culture was first inoculated on a potato dextrose agar (PDA) medium plate and incubated at a constant temperature of 23 °C in the dark for 10 to 14 days, allowing the mycelium to grow fully. Subsequently, well-developed mycelium was selected and transferred to a pre-prepared original seed medium, consisting of 84% wheat, 10% cottonseed hulls, 4% sawdust, 1% lime, and 1% gypsum, for further cultivation at 20 °C in the dark for 25 to 30 days [23,24].

In a temperature-controlled incubation room maintained at 20 °C, the mycelium continued to grow in the primary culture medium until it fully colonized the surface. The next step was to transfer the colonized mycelium to a new bulk substrate, which consisted of 84% sawdust, 14% cottonseed hulls, 1% lime, and 1% gypsum. The inoculated medium was then cultivated in a cultivation chamber maintained at 20 °C in the dark for 45–50 days until the mycelium fully covered the medium. In the final stage, when the mycelium was mature, the cultivation bag was opened and the environmental conditions were adjusted to an appropriate temperature (23 °C) and a relative humidity range of 70–80%, to induce the formation and growth of fruiting bodies. The process continued for 5–7 days until fruiting bodies had successfully formed [23,25,26,27,28].

### 2.2. Fruiting Body Tissue Culture and Mycelium Collection

The fruiting body tissue was excised and inoculated on PDA plates, and then it was incubated in the dark at 23 °C for 16 days. After incubation, the mycelium was harvested and flash-frozen in liquid nitrogen for subsequent genome sequencing and chromosome-level assembly [29,30,31,32].

### 2.3. Genomic DNA Extraction

The genomic DNA of strain ZZ1 was extracted using the Ezup Column Fungi Genomic DNA Extraction Kit provided by Sangon Biotech Co., Ltd. (Shanghai, China; https://www.sangon.com/; accessed on 1 December 2024). The concentration and quality of the extracted DNA were analyzed using a UV-Vis spectrophotometer (IMPLEN) to ensure that the purity and concentration met the requirements for genome sequencing [33].

### 2.4. Genome Sequencing and Raw Data Preprocessing

The Illumina sequencing platform was used to sequence the ZZ1 genome, resulting in high-quality sequencing data with sufficient coverage. Fastp software (V 0.11.2) was used to collect key metrics, including quality scores of the raw sequencing data, and generate a base quality score distribution plot. To ensure the integrity of downstream analyses, the raw data underwent strict quality filtering to produce high-quality clean data. Additionally, FastQC software (V 0.11.2) was used to perform a visual quality assessment of the sequencing data, further confirming the reliability of the clean data (https://www.bioinformatics.babraham.ac.uk/projects/fastqc/; accessed on 1 December 2024).

### 2.5. Nanopore Sequencing Data Processing

The quality control of raw nanopore sequencing data is crucial to achieve optimal results. Through k-mer analysis, critical information such as the genome size, heterozygosity, and proportion of repetitive sequences can be efficiently derived.

### 2.6. Genome Splicing and Completion

Canu (https://github.com/marbl/canu, accessed on 1 December 2024) was used to assemble Nanopore (https://pmc.ncbi.nlm.nih.gov/articles/PMC10333861/, accessed on 1 December 2024) sequencing data, for a preliminary genome assembly [34]. Following this, Illumina sequencing data were incorporated, and GapFiller (V 1.11) (https://www.baseclear.com/microbiome-analysis-bioinformatics/, accessed on 1 December 2024) was used for gap filling [35,36], enhancing the genome integrity. Additionally, Pilon was used for polishing to correct errors and small indels that occurred during the assembly process, thereby further improving the genome’s accuracy. BUSCO (V 1.0) was used to verify the genome assembly and assess the gene set completeness [37].

#### 2.6.1. Gene Component Analysis

GeneMark-ES (https://exon.gatech.edu/GeneMark/, accessed on 1 December 2024) and GlimmerHMM (https://ccb.jhu.edu/software/glimmerhmm/, accessed on 1 December 2024) were used for de novo gene prediction using the assembled genome, while RepeatModeler was used for the de novo identification of repetitive sequences. RepeatMasker (https://www.repeatmasker.org/, accessed on 1 December 2024) was then used to identify the locations and frequencies of different types of repetitive sequences in the genome.

#### 2.6.2. Gene Annotation

To comprehensively analyze the functional characteristics of protein sequences, we utilized NCBI BLAST (https://blast.ncbi.nlm.nih.gov/Blast.cgi, accessed on 1 December 2024) to perform comparisons against multiple authoritative databases [38]. Specifically, we compared the protein sequences against the Conserved Domain Database (CDD), Eukaryotic Orthologous Gene Cluster Database (KOG), Non-redundant Protein Sequence Database (N-R), and Protein Family Database (PFAM) (http://pfam.xfam.org/, accessed on 1 December 2024) to obtain functional annotations [39].

### 2.7. Determination of Mating Site

By using the mating-type genes from other *Pholiota* strains as reference sequences, DIAMOND was used to perform sequence alignment, to analyze the mating-type loci of ZZ1.

### 2.8. Identification of CAZymes

This study used CAZy (Carbohydrate-Active Enzymes Database) to annotate carbohydrate-active enzymes in the protein sequences of the gene set. The CAZy database is a specialized resource for carbohydrate-active enzymes, including the enzyme families involved in carbohydrate degradation, modification, and biosynthesis. It is organized into five main categories: glycoside hydrolases (GHs), glycosyltransferases (GTs), polysaccharide lyases (PLs), carbohydrate esterases (CEs), and auxiliary activities (AAs). Additionally, the CAZy database includes carbohydrate-binding modules (CBMs), which play an important role in carbohydrate recognition and binding. To obtain functional annotations for carbohydrate-active enzymes in the protein sequences of the gene set, we employed HMMER3 (http://hmmer.org/, accessed on 1 December 2024) software to align the protein sequences with hidden Markov models (HMMs) in the CAZy database [40], with an E-value threshold of <1 × 10^−5^.

### 2.9. Pan-Genome Analysis

Several methods are employed at the national and international levels for fungal functional characterization in various species. These include biochemical detection; genomic analysis; metabolomic analysis; resistance testing and enzyme activity assays; traditional morphological and physiological assessments; high-throughput screening; functional verification; and the use of molecular markers. However, the application of genomic analysis methods is relatively limited, primarily due to the significant variability in functional genes across different strains of the same species, which is related to the concept of the bacterial pan-genome [41]. This is the total set of genes found across different strains of a bacterial species [42], which can be divided into the core genome and the dispensable genome. The core genome comprises genes shared by more than 95% of the strain genomes within the same species, and that are essential for the species’ survival. Given that there are variations in functional genes among different strains of the same genus, it is important that we examine the genetic differences and diversity among strains or isolates [43].

In this study, we obtained genomic data from four strains (*P. nameko* and related strains) in the NCBI RefSeq database and conducted a pan-genome analysis. We employed OrthoFinder2 to identify both core and dispensable genes among the sequenced strains for further in-depth analysis. Homologous genes found in all samples were defined as core genes [44]. After excluding core genes, we identified dispensable genes, defined as those unique to a specific sample. Dispensable genes, along with shared genes, formed the pan-genome. As shared and unique genes are likely associated with the shared and unique characteristics of the samples, these served as the basis for our investigation of the functional differences among the strains. This approach allowed us to explore the genetic differences among the strains at the genomic level [45,46].

## 3. Results

### 3.1. Cultivation and Strain Isolation

**Figure 1:**
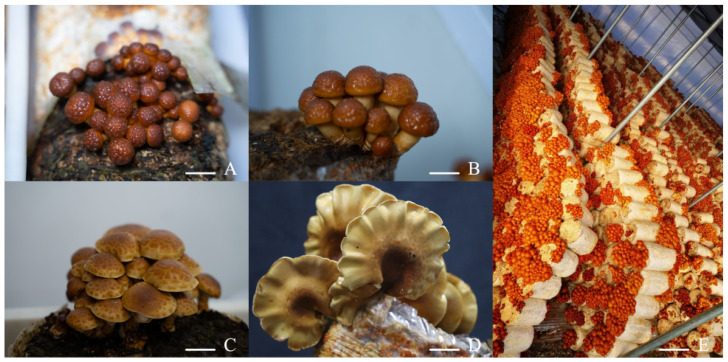
The cultivation conditions and primary processes involved in the development of the fruiting body of *P.nameko* ZZ1. (**A**) Primordium stage. (**B**) Growth stage. (**C**) Cap expansion stage. (**D**) Full maturity stage. (**E**) Mushroom cultivation in a greenhouse. Scale Bars = 3 cm.

The cultivation conditions and primary processes used in the development of the fruiting body of *P. nameko* ZZ1 (Shaanxi, China, as studied in our laboratory) are presented in Figure 1. During the primordium stage (Figure 1A), a monokaryotic mycelium pairs with another that is compatible to form a dikaryotic mycelium, which subsequently undergoes reproduction. The growth stage follows primordium formation (Figure 1B), during which continuous growth occurs and the fruiting body continues to develop until a complete cap is formed. In the Figure 1C stage, the fruiting body reaches 70–80% maturity. Then, the maturity stage (Figure 1D) marks the point when the fruiting bodies are fully mature and spores are collected and plated. Figure 1E shows the stage when the entire mushroom is fully visible.

### 3.2. Genome Sequence Assembly

**Figure 2:**
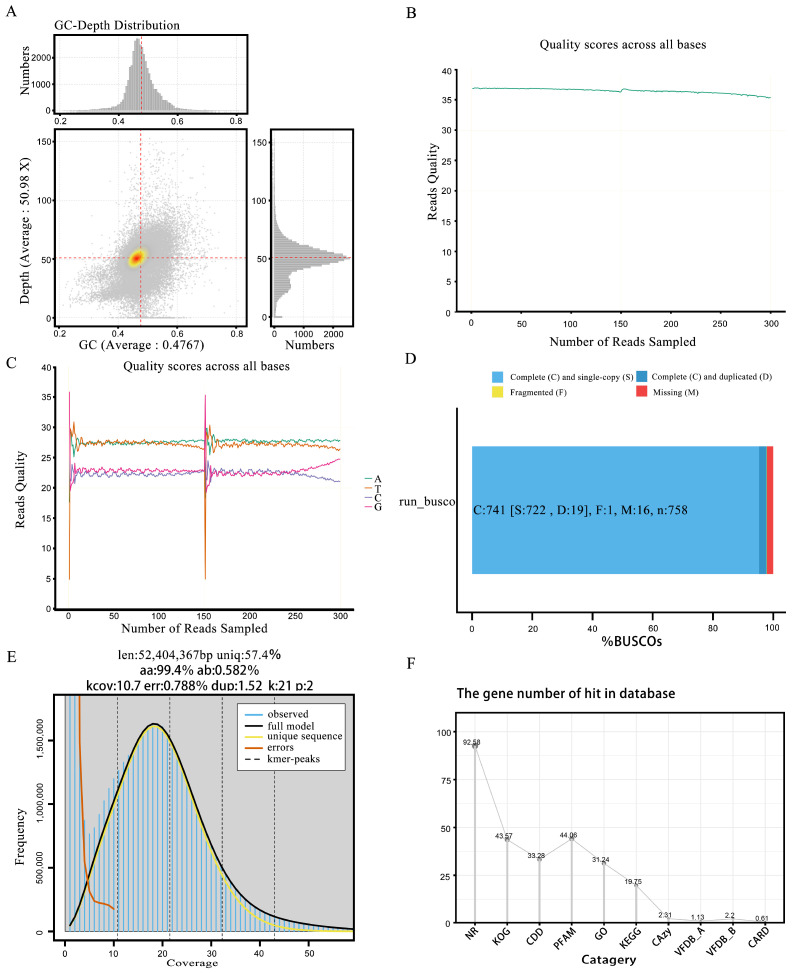
Comprehensive genomic quality and content assessment summary chart. (**A**) Genome gc-depth distribution chart. (**B**) Base quality distribution chart. (**C**) Single-sample sequencing base content distribution chart. (**D**) The BUSCO evaluation distribution bar chart. The bar chart shows the proportion of each component. (**E**) The survey assesses the k-mer distribution plot. The blue bars in the figure represent actual observed values. (**F**) Line chart of database annotation ratio.

During the Illumina sequencing process, we initially utilized Fastp software to perform a quality assessment and statistical analysis of the raw data. The results revealed 3,280,402,500 base pairs, with an average sequence length of 150 base pairs. The data were reassembled into 33 contigs (scaffolds). Among these, 3,219,066,634 bases had a quality score of Q20 or above, accounting for 98.13% (Table 1), while 3,129,235,413 bases had a quality score of Q30 or above, representing 95.39%. Additionally, the GC content of this genome was 45.25% (Figure 2A). To more intuitively represent the data quality, we used GenomeScope software (V 2.0) to generate distribution charts of the base quality (Figure 2B) and single-sample base content (Figure 2C).

For accurate information analysis, we rigorously filtered the adapter sequences and low-quality sequences from the original data to obtain clean data. Following quality control, the size of the dataset was 3,172,382,043 bp, with the average sequence length reduced to 145.36 bp. Among these data, 3,112,232,462 bases had a quality score of Q20 or above, representing 98.10%, while 3,023,938,859 bases had a quality score of Q30 or above, accounting for 95.32%. The clean read rate reached an impressive 99.795%, with 21,824,452 reads in the sample. Additionally, the GC content after quality control was 45.06% (Table S1).

We utilized Canu software to assemble third-generation long-read sequencing data, integrating short-read second-generation sequencing data for additional support. Following assembly, we employed GapFiller to address any remaining gaps in the scaffold. Moreover, we applied PrInSeS-G (https://updeplasrv1.epfl.ch/prinses/; accessed on 1 December 2024) [47] software to enhance the sequence accuracy and completeness by correcting base errors and small-fragment insertions and deletions (indels) during assembly. Subsequently, we conducted three rounds of iterative polishing using Pilon until no significant errors were detected through read alignment. Once finalized, we adjusted the sequence orientation based on the minimum GC skew score, using this as the transcription start site. We then compared the results with the replication initiation protein database. Simultaneously, the sequencing depth and coverage were calculated and compared with the NT library to obtain species classification results.

**Figure 3:**
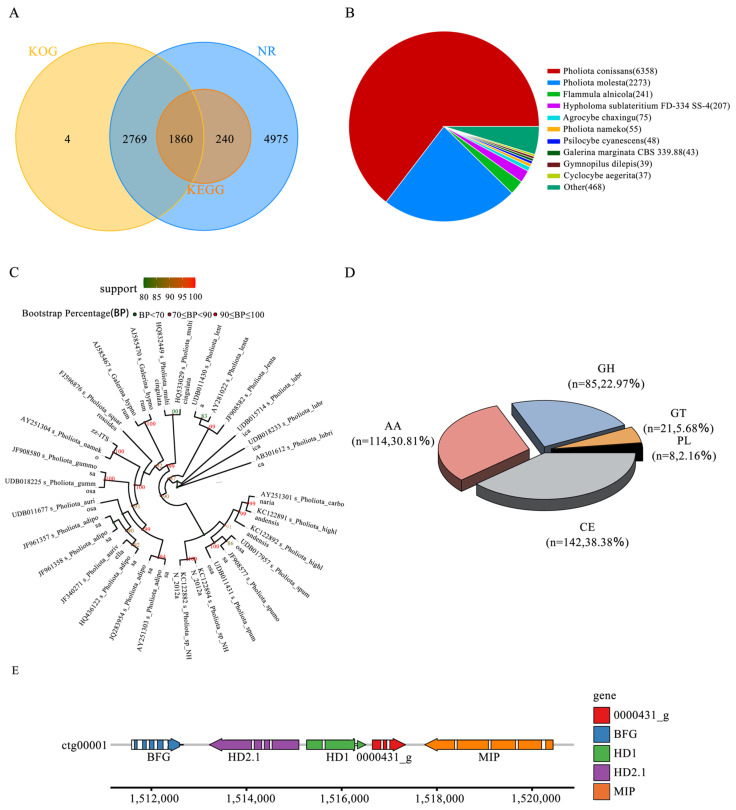
Gene annotation and homologous species distribution. (**A**) Venn diagram of gene annotations from databases. (**B**) Distribution pie chart of homologous species. Each sector in the chart represents a species, and the larger the area of the sector, the greater the number of sequences aligned to that species. (**C**) ITS-based circular phylogenetic tree. (**D**) CAZy Class classification. (**E**) *A*-mating-type site of *P. nameko* ZZ1.

We used BUSCO, a tool designed to assess the genome assembly quality, to evaluate its accuracy and completeness. BUSCO constructed a set of single-copy genes from several major evolutionary lineages using the OrthoDB (http://cegg.unige.ch/orthodb, accessed on 1 December 2024) database and compared these sets to the assembled genome. The results showed that over 80% were complete, indicating high genome assembly completeness (Figure 2D). Furthermore, a single K-mer coverage peak was observed (Figure 2E), indicating a heterozygosity rate of 0.51%. These findings, along with the other analytical results (Figure 2F and Figure 3A), confirmed that ZZ1 is a dikaryotic strain.

### 3.3. Genomic Analysis

In this study, we evaluated the genome size of *P. nameko* ZZ1 and found it to be comparable to that of other species in the genus *Pholiota*. Specifically, the genome is slightly larger than that of *P. micraspora* but smaller than those of *P. molesta*, *P. adiposa*, and *P. conissans*. Notably, the GC content of ZZ1 is lower than those of most other fungal species. Regarding the genome structure, we identified a total of 10,707 repeat sequences, which account for 15.05% of the total genome length. Among these, Gypsy-type long terminal repeat (LTR) sequences constitute the largest proportion, reaching 4.59%. Additionally, 0.54% of the repetitive elements are simple repeat sequences, while 0.13% are DNA transposons of the TcMar-Sagan family (Table S2). In our gene prediction, we identified 87,544 gene models from the ZZ1 genome, including 9872 known, 70,570 newly predicted, 2130 pseudo, and 4972 unclassified genes. The average sequence length of these gene models is 1753 bp, with 15,697,970 bp gene bases in the CDS region (Table S3).

We conducted an annotation analysis to gain insights into these genes’ functional properties. In the NR and KOG databases, 9844 (92.58%) and 4633 genes (43.57%) were annotated, respectively (Table S4), while based on protein similarity, 4685 genes (44.06%) were annotated in the PFAM database. Additionally, we utilized BUSCO software to assess the completeness of the ZZ1 genome assembly and gene prediction, finding that the completeness was high at 97.76% (Table S5). In summary, this study successfully generated a high-quality ZZ1 genome, which exhibited favorable characteristics in terms of size, structure, gene prediction, and annotation. This work provides a foundation for subsequent functional gene mining, genetic variation analysis, and comparative genomics.

### 3.4. Phylogenetic Analysis

To thoroughly investigate the evolutionary relationships between ZZ1 and ten other representative basidiomycete strains, we employed the NCBI BLAST method and compared our data with the NR database. Our objective was to elucidate the degree of similarity between the transcript sequences of the species under study and those of closely related species, while acquiring functional information regarding homologous sequences. In the alignment diagram we constructed, each sector represents a species, and its size reflects the number of sequences aligned to that species. Notably, we concentrated on the ITS sequence, which is situated between the 18S, 5.8S, and 28S rRNA genes of the fungus, encompassing both ITS 1 and ITS 2. ITS sequences are characterized by high conservation among fungi, and as they are subject to little natural selection pressure, they can accommodate many mutations throughout their evolution, meaning they exhibit a rich array of sequence polymorphisms in most eukaryotes.

We used the BLAST tool to align the ITS sequences predicted by the genes with the ITS sublibrary in the NCBI-NT database (identity > 95). Subsequently, we selected the 30 ITS sequences with the highest identity, used the mafft software for the multiple alignment of the sequences, and performed shearing processing. Finally, we constructed a phylogenetic tree using the FastTree, iqtree, and raxml software. In our phylogenetic analysis, we identified 9844 single-copy orthologous genes in 30 fungal species. These genes cover *Pholiota conissans*, *Pholiota molesta*, *Flammula alnicola*, *Hypholoma sublateritium* FD-334 SS-4, *Agrocybe chaxingu*, *Pholiota nameko*, *Psilocybe cyanescens*, *Galerina marginata* CBS 339.88, *Gymnopilus dilepis*, *Cyclocybe aegerita*, and other species (Figure 3B). Through the phylogenetic tree constructed using the maximum-likelihood method (Figure 3C), we could clearly observe that all Lepidoptera species were clustered on one evolutionary branch, with *P. nameko* ZZ1 and *P. nameko* located close to one another, further confirming their genetic relationship.

### 3.5. CAZymes Analysis

Carbohydrate-active enzymes (CAZymes) are a core gene family within fungal genomes and are essential for lignocellulose degradation, as well as various biological processes, including developmental regulation and the stress response [48]. This study employed HMMER3 software (V 3.1b1) for a comprehensive comparison and analysis of the target genome’s protein sequences against the CAZy database, applying stringent screening criteria (E-value < 1 × 10^−5^) to ensure the accuracy and reliability of the annotation. In the genome of *P. nameko* ZZ1, we identified 246 enzymes with specific functions, categorized into 68 distinct CAZyme categories. Specifically, these enzymes comprised 114 auxiliary active enzymes (AAs), 142 carbohydrate esterases (CEs), 85 glycoside hydrolases (GHs), 21 glycosyltransferases (GTs), and 8 polysaccharide lyases (PLs) (Figure 3D). Further analysis revealed that the 114 AA-type proteins in *P. nameko* ZZ1 are broadly distributed across multiple families, ranging from AA1 to AA12. Among these, the AA3 and AA9 categories are particularly significant, as their proteins are directly involved in cellulose and hemicellulose degradation. Additionally, enzymes associated with lignin degradation, such as laccases and peroxidases, fall within the AA1 and AA2 families, respectively. Through an analysis of the CAZy professional database, we annotated 11 laccase genes in this strain, which are crucial in the degradation of microbial cell wall components [49].

### 3.6. Identification of Mating-Type Sites

This study utilized the *A*-mating-type, derived from the *Grifola grifola* strain HM62, as the reference sequence for an in-depth gene alignment analysis. The results demonstrated a clear correspondence between the reference sequence and four genes (ctg0001_0000428_ t to ctg00001_0000432_t) from 1511585 to 1520441 on the ctg00001 chromosome, with a total length of 8857 bp. Notably, two homeodomains (HDs) were identified among the loci analyzed, namely HD1 and HD2. The latter corresponds to the gene ctg00001_0000429_t, with position coordinates ranging from 1513218 to 1515100 and a reverse transcription direction (i.e., − strand). Meanwhile, HD1 corresponds to the gene ctg00001_0000430_t, with position coordinates from 1515264 to 1516507 and a forward transcription direction (i.e., +strand). In summary, through precise alignment analysis, this study not only clarified the correspondence between the *A*-mating-type reference sequence and specific loci on the ctg00001 chromosome, but also elucidated the relationship between the two key homeotic domains (HD1 and HD2) [50]. The detailed location and transcription direction provide important references for subsequent functional studies and genetic analyses (Figure 3E).

### 3.7. Genome Functional Annotation

**Figure 4:**
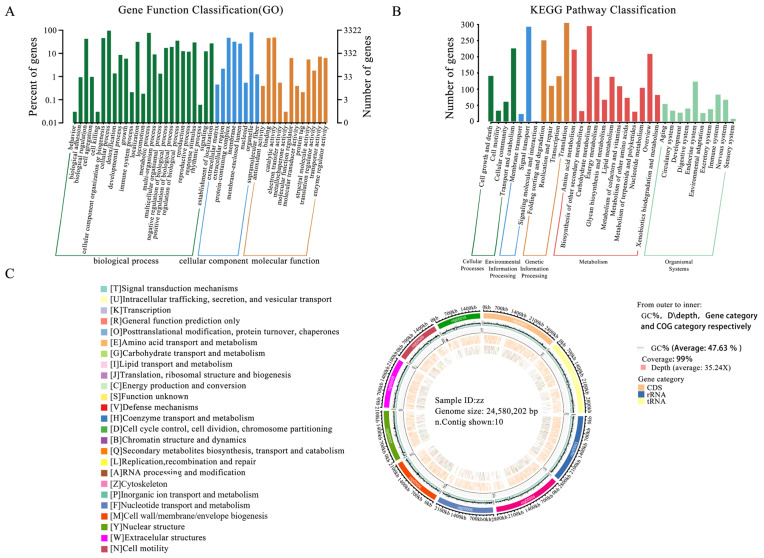
Comprehensive display of gene functional annotation and genome features. (**A**) GO annotation distribution bar chart. The horizontal axis represents the second-level GO categories, while the vertical axis shows the number of genes in each category (right) and their percentage of the total annotated genes (left). Different colors represent different orthologs. (**B**) KEGG Pathway classification bar. The horizontal axis represents the names of the metabolic pathways, while the vertical axis shows the number of genes annotated to each pathway. (**C**) Genome circos plot display. The circos plot displays, from the outside to the inside, the GC content, sequencing depth, gene elements, and COG functional annotation.

To achieve a comprehensive functional annotation of the ZZ1 strain genome, we utilized nine databases (including NT, NR (http://ncbi.nlm.nih.gov/, accessed on 1 December 2024), COG [51] (https://www.ncbi.nlm.nih.gov/COG/, accessed on 1 December 2024), Swiss-Prot, TrEMBL, PFAM, CDD [52] (https://www.ncbi.nlm.nih.gov/cdd/, accessed on 1 December 2024), GO (http://www.geeontology.org, accessed on 1 December 2024), and KEGG [53] (http://www.kegg.jp, accessed on 1 December 2024) to perform sequence similarity analysis on 10,633 genes (Table S4). Our functional annotation of protein-coding genes in strain ZZ1 revealed significant functional diversity across the databases. We annotated 3322 genes in the GO database, with classification by biological process being the most prominent (Figure 4A). Additionally, 2100 genes were identified in the KEGG database, and these were associated with five pathway types, with metabolic pathways being most abundant (Figure 4B). To present the genome characteristics more intuitively, we employed Circos software (V 0.69-9) for a comprehensive visualization of the genome, encompassing information such as the GC content, sequencing depth, gene element content, and COG functional distribution (Figure 4C).

### 3.8. Pan-Genome Analysis Annotation

**Figure 5:**
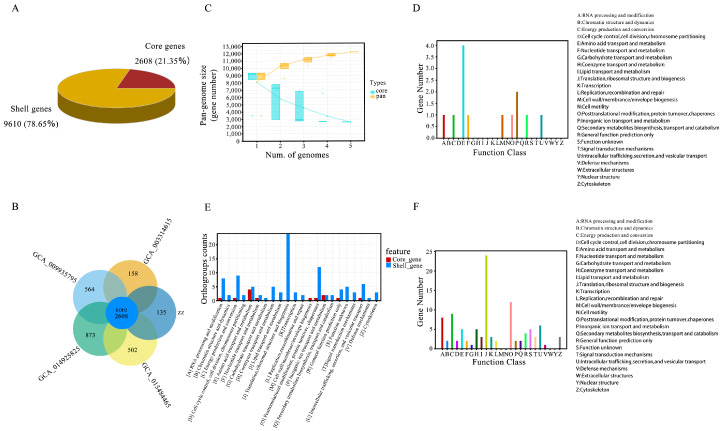
Comparative analysis and functional distribution of core and shell genes. (**A**) Pie chart of homologous genes categories. (**B**) Flower plot of homologous genes. (**C**) The accumulation boxplot of core/pan genes. (**D**) The distribution of core genes across different functional gene categories. (**E**) Comparative gene plot of core genes and shell genes. (**F**) The distribution of shell genes across different functional gene categories.

We conducted a large-scale genome comparison analysis of five strains of the same genus. We obtained 12,218 high-quality genomes when using genome completeness thresholds of >90%, 15%, and 1% as the quality filters (Table 2). Among these, 2608 core genes were identified, accounting for 21.35% of the total genes, while 9610 shell genes, corresponding to strains with 15% to <90% completeness, accounted for 78.65% (Figure 5A). The analysis revealed that *P. molesta* has the largest number of genomes within the genus *Pholiota*, with 3481 core genes and 873 dispensable genes. In comparison, *P. nameko* has 2743 core genes and 135 dispensable genes; *P. adiposa* has 3174 core genes and 564 dispensable genes; *P. microspora* has 2766 core genes and 158 dispensable genes; and *P. conissans* has 3110 core genes and 502 dispensable genes (Table 3). Strong evidence of the same pan-genome structure was found across the five fungal species, indicating that their pan-genomes comprise core genes (Figure 5B). To visually represent the pan-genome characteristics of the genus *Pholiota*, a cumulative boxplot of core and pan-genes was created. This analysis demonstrated that the number of pan-genomes for each species continued to increase with the addition of genomes, while the number of core genomes exhibited both increases and decreases (Figure 5C). This pattern is characteristic of an open pan-genome, suggesting that the strains exhibit high genetic diversity.

In the functional categorization of pan-genome genes, the core genes associated with amino acid transport and metabolism were the most numerous, followed by genes involved in inorganic ion transport and metabolism (Figure 5D). For shell genes, the categories involved in translation, ribosomal structure, and biosynthesis had the highest gene counts, followed closely by those involved in post-translational modification (Figure 5E). A comparative analysis of the number of genes in the core and shell categories, represented by the letters A to Z, was also conducted. As illustrated in Figure 5, the core genes associated with amino acid transport and metabolism were predominant, suggesting that this function is both widespread and conserved across the five strains. The large number of shell genes related to translation, the ribosomal structure, and biosynthesis suggests a significant functional variability among strains. These genes are likely linked to environmental adaptability and species specificity, showing lower conservation and a stronger association with the specific ecological niches of these species (Figure 5F).

### 3.9. Virulence Factors and Resistance Factors

We conducted an in-depth study of the virulence factors and drug resistance mechanisms of strain ZZ1. Using the BLAST algorithm, we compared the gene protein sequences against the VFDB (Virulence Factor Database) [54] (https://www.mgc.ac.cn/VFs/, accessed on 1 December 2024) and CARD [55] (https://card.mcmaster.ca/, accessed on 1 December 2024) (Comprehensive Antibiotic Resistance Database), integrating gene data with their functional annotations for virulence factors and drug resistance genes to achieve comprehensive annotation results [56]. The virulence factors identified were classified into two groups: SetA and SetB. In SetA, 35 distinct virulence factors were identified, primarily involved in immune regulation, mucosal adherence, motility, antibiotic resistance, and related functions. In SetB, 87 virulence factors were identified. The most frequent were pyoverdine (six occurrences), LPS (four occurrences), and factors related to PDIM (quinone dimyristate) and PGL (phenolic glycolipids), each found four times.

## 4. Discussion

The genus *Pholiota* (*Pholiota Kummer*) was established by Kummer in 1871 and is distributed worldwide, belonging to the family Mycenaceae. In China, this genus comprises 63 species and 2 varieties, which are found across 27 provinces and autonomous regions. Although *Pholiota* is an important taxon within Mycenaceae, its genome has not been extensively studied. This study presents, for the first time, the complete genome of *P. nameko*, a member of the genus *Pholiota*. The comprehensive analysis demonstrates the high quality of the genome assembly. Overall, our findings provide a foundation for future cultural, nutritional, and medicinal research on *Pholiota*.

In this study, we obtained the *P. nameko* genome, which is 54.28 Mb, 35.1 Mb, 66 Mb, and 44 Mb greater than that of four other Lepidoptera species (*Pholiota adiposa*, *Pholiota microspora*, *Pholiota molesta*, and *Pholiota conissans*, respectively). The *P. nameko* ZZ1 genome is sized at 24.58 Mbp, is composed of 33 contigs, and has an N50 of 2.11 Mb and a GC content of 42.25%. In the *P. nameko* ZZ1 genome, we predicted a total of 87,544 gene models, which includes 9872 known, 70,570 newly predicted, 2130 pseudo, and 4972 unclassified genes. The average sequence length of these gene models was 1753 bp, and there were 15,697,970 gene bases in the coding sequence (CDS) region. When compared to strains of other species within the same genus, the genome of *P. nameko* ZZ1 was slightly larger than that of *P. microspora*, but smaller than the genomes of *P. molesta*, *P. adiposa*, and *P. conissans*. Additionally, the GC content of *P. nameko* ZZ1 was lower than that of most Lepidoptera species. Phylogenetic analysis confirmed that *P. nameko* should be classified within the genus *Pholiota*. We identified 246 enzymes with specific functions in the *P. nameko* ZZ1 genome, belonging to 68 categories of carbohydrate-active enzymes (CAZymes) [57] (http://www.cazy.org/, accessed on 1 December 2024). The identification of CAZymes in *P. nameko* ZZ1 indicates that this strain possesses a robust lignocellulose-degrading ability, which includes 114 auxiliary active enzymes (AAs), 142 carbohydrate esterases (CEs), 85 glycoside hydrolases (GHs), 21 glycosyltransferases (GTs), and 8 polysaccharide lyases (PLs). AAs are typically involved in redox reactions and can influence the metabolic pathways of fungi, aiding in energy acquisition and adaptation to environmental changes. For instance, they play a role in the metabolism of amino acids, lipids, and other small molecules, and they regulate the growth and differentiation of fungal cells [58].

Carbohydrate enzymes (CEs) catalyze the hydrolysis of ester bonds in carbohydrates, facilitating the decomposition of complex sugars such as cellulose and lignin, and thereby providing essential carbon sources for fungi. This process is critical for nitrogen fixation, nutrient acquisition, and overall fungal growth in natural environments [59,60]. Glycoside hydrolases (GHs) hydrolyze glycosidic bonds, breaking down polysaccharides such as cellulose, starch, and pectin to supply fungi with necessary sugars. These carbohydrate nutrients are vital for fungal growth on complex substrates, whether saprophytic or parasitic, as they enable the digestion of plant material and other organic matter. Glycosyltransferases (GTs) play a crucial role in carbohydrate synthesis by catalyzing the transfer of carbohydrate units to form new glycoside chains [61,62]. They are involved in the synthesis and glycosylation modification of cell wall polysaccharides, contributing to biological macromolecules’ construction and repair, such as by strengthening fungal cell walls. Pectinases and ligninases (PLs) catalyze polysaccharide cleavage, particularly breaking down complex sugars such as pectin and starch to release accessible sugars. PLs play a significant role in fungal nutrient acquisition, host plant infection, and adaptation to environmental stress [63]. Collectively, these enzymes facilitate nutrient acquisition, cell wall synthesis, environmental adaptation, and fungal growth and development by aiding in complex organic substances’ decomposition and crucial biological macromolecules’ synthesis and modification [64,65].

In this study, we conducted a pan-genome analysis on five closely related *Pholiota* strains, yielding extensive genomic information. The results revealed significant genetic differences among these strains, underscoring the importance of pan-genome analysis in elucidating species diversity [66]. In the findings, the genus *Pholiota* exhibited typical open pan-genome characteristics, reflecting the high genetic diversity and extensive gene combinations among different strains. This diversity highlights the adaptability and complexity of species evolution within the genus. Core genome analysis demonstrated that amino acid transport and metabolism are common and conserved functional categories across these strains, suggesting that these functions are crucial for the survival of the genus *Pholiota* and may play a key role in maintaining essential life activities and adapting to various environments [67]. Additionally, inorganic ion transport and metabolism emerged as prominent core functions, emphasizing the conservation of these metabolic pathways among different strains. In contrast to core genes, shell genes exhibited greater diversity, accounting for the highest proportions of genes in functional categories such as translation, ribosome structure, and biosynthesis. This genetic diversity may relate to environmental adaptability and specific functions among strains, indicating that these genes could be subject to selective pressures from environmental conditions, increasing variability among different strains [68]. These genes’ diversity enhances the ability of *Pholiota* to adapt to changing living environments, and this variability may be closely linked to the competitive survival of strains in specific habitats. The functional differentiation we found not only reflects the balance between gene conservation and variability among *Pholiota,* but also indicates that these strains possess highly flexible genome structures, enabling them to adapt to various environmental challenges. This finding offers significant insights into the ecological adaptability and potential functional diversity of *Pholiota*, while also highlighting directions and foundations for future research on functional genes.

We also identified and characterized repetitive sequences and gene models within the *P. nameko* ZZ1 genome structure. Our results revealed 10,707 repetitive elements, which constitute 15.05% of the genome’s total length. Among these repetitive sequences, the long terminal repeat (LTR) family Gypsy comprises the highest proportion, at 4.59%. Additionally, some repetitive elements are simple repeat sequences, accounting for 0.54% of the genome, while the DNA transposon TcMar-Sagan represents 0.13%. In gene prediction, we identified 87,544 gene models from the *P. nameko* ZZ1 genome, which includes both known and newly predicted genes. Specifically, 9872 are known genes, while 70,570 are newly predicted, suggesting the significant presence of previously unrecognized potential functional genes. Furthermore, we identified 2130 pseudogenes and 4972 unclassified genes. These unclassified and putative genes provide a foundation for further research and may offer new insights related to species characteristics. The average sequence length of all gene models was 1753 bp, indicating a relatively uniform gene length and moderate complexity. We determined that there were 15,697,970 bp bases in the coding sequence (CDS) region of the gene, reflecting the relatively compact gene-coding characteristics of the *P. nameko* ZZ1 genome.

Finally, we conducted an in-depth comparison of the *A*-mating-type reference sequence and the ctg00001 chromosome of *Grifola frondosa* HM62, yielding significant findings. These offer a detailed molecular basis for understanding the genetic characteristics of this strain within the mating-type gene region [69], particularly regarding the positioning and directionality of the HD. This research establishes a crucial foundation for genetic analyses and functional investigations of the mating types of *Grifola frondosa*. By elucidating the specific locations and transcriptional directions of these two homeodomains, future studies can further examine their functional roles in mating signaling or investigate their expression patterns at various developmental stages. Such research is vital to enhance our understanding of the mating biology of *Grifola frondosa* and may inform strategies for artificial breeding and genetic improvement. Additionally, repeat sequence analysis and mating-type loci can be utilized for strain identification and the development of molecular markers in breeding. The *P. nameko* genome provides a basis for cultivation as well as nutritional and medicinal research on *Pholiota*.

## 5. Conclusions

*Pholiota nameko* is a rare mushroom. In this study, we performed whole-genome sequencing and a fine mapping analysis of the *P. nameko* strain ZZ1 for the first time using Illumina and Nanopore sequencing technologies. This provided important clues for studying the mating loci and pan-genome of this fungus, while also offering a comprehensive genetic blueprint for this commercially and nutritionally valuable mushroom species. The genomic analysis revealed diverse carbohydrate-active enzymes (CAZymes), highlighting the significant role played by *P. nameko* in lignocellulose degradation and further emphasizing the strain’s ability to break down complex polysaccharides, which is crucial for nutrient acquisition and environmental adaptability. However, although we have identified the presence of these enzymes at the genomic level, the specific expression of enzyme activity and other biological properties still needs to be experimentally validated. In future studies, we plan to conduct in vitro experiments to further confirm the function and activity of these enzymes to verify the results predicted by bioinformatics. The phylogenetic analysis demonstrated a high degree of genetic collinearity between *Pholiota nameko* and closely related strains. The pan-genome analysis of five *Pholiota* strains revealed significant genetic diversity, indicating an open pan-genome structure. That genetic variability is crucial for understanding the adaptability and evolution of species within the genus. The comprehensive functional annotation of the *P. nameko* genome, supported by multiple databases, elucidated its fundamental biological processes and metabolic pathways, laying the foundation for future studies in molecular breeding, cultivation technology, and the nutritional and medicinal properties of *P. nameko*. Additionally, it provides a critical basis for enhancing *P. nameko* cultivation, improving strain selection, and harnessing its potential benefits in agricultural and pharmaceutical applications.

## Figures and Tables

**Table 1:** Post-quality control data statistics table.

Characteristics	*P. nameko* ZZ1
Total Reads Count	21,869,350
Total Bases Count (bp)	3,280,402,500
Average Read Length (bp)	150
Q20 Bases Count (bp)	3,219,066,634
Q20 Bases Ratio	98.13%
Q30 Bases Count (bp)	3,129,325,413
Q30 Bases Ratio	95.39%
GC content	45.25%

**Table 2:** Gene classification.

Genes	Strains	Number
Core genes	(90% ≤ strains ≤ 100%)	2608
Shell genes	(15% ≤ strains < 90%)	9610
Cloud genes	(1% ≤ strains < 15%)	0
Total genes	(0% ≤ strains ≤ 100%)	12,218

**Table 3:** The pan-genome of five *Pholiota* genus strains in NCBI.

Latin Name	GeneBank	Pan	Core	Dispensable
*Pholiota nameko*	PQ839732	2743	2608	135
*Pholiota adiposa*	GCA_009935795.1	3174	2608	564
*Pholiota microspora*	GCA_003314615.1	2766	2608	158
*Pholiota molesta*	GCA_014925825.1	3481	2608	873
*Pholiota conissans*	GCA_015484465.1	3110	2608	502

## Data Availability

The complete genome sequence data of the species will be registered and stored on the GenBank (https://www.ncbi.nlm.nih.gov/genbank/, accessed on 1 December 2024). Specimens are deposited at the College of Life Sciences, Yan’an University, Yan’an 716000, Shaanxi Province, China. All the experimental data related to this work were included in the manuscript, figures, or Supplementary Information/dataset(s).

## Supplementary Materials

The following supporting information can be downloaded at: https://www.mdpi.com/article/10.3390/jof11020112/s1, Table S1: Post-QC Data Statistics; Table S2: Repeat sequence prediction status; Table S3: Genetic Prediction Results; Table S4: Gene annotation ratio statistics; Table S5: BUSCO Completeness Assessment.
