# Supplementary material for: Whole-Genome Sequencing and Fine Map Analysis of Pholiota nameko
Source: J Fungi (Basel). 2025 Feb 3;11(2):112. doi: 10.3390/jof11020112 (PMC11856836; doi:10.3390/jof11020112)
Supplement: Supplementary file 1 [file jof-11-00112-s001.zip › jof-3437957-supplementary.pdf]

**Table S1.** Post-QC Date Statistics.

| Characteristics | ***P.nameko ZZ1*** |
|---|---|
| Total Reads Count(#) | 21824452 |
| Total Bases Count(bp) | 3172382043 |
| Average Read Length(bp) | 145.36 |
| Q20 Bases Count(bp) | 3112232462 |
| Q20 Bases Ratio(%) | 98.10% |
| Q30 Bases Count(bp) | 3023938859 |
| Q30 Bases Ratio(%) | 95.32% |
| GC content(%) | 45.06% |
| Clean Reads(%) | 99.795% |
| Low Quality(%) | 0.0% |
| Too Many N(%) | 0.196% |
| Too short(%) | 0.009% |

**Table S2.** Repeat sequence prediction status.

| Repeat Family | Region Count | Base Count | Min Length | Max Length | Average Length | Percentage In Genome(%) |
|---|---|---|---|---|---|---|
| Unknown | 2103 | 1579394 | 26 | 23688 | 751.02 | 3.98 |
| Simple repeat | 4972 | 212698 | 6 | 321 | 42.78 | 0.54 |
| Low complexity | 700 | 36815 | 13 | 170 | 52.59 | 0.09 |
| LTR:Gypsy | 717 | 1820595 | 36 | 53387 | 2539.18 | 4.59 |
| LTR:Copia | 399 | 488756 | 26 | 9990 | 1224.95 | 1.23 |
| LINE:Tad1 | 773 | 1143789 | 37 | 7217 | 1479.68 | 2.89 |
| LINE:RTE-X | 31 | 109563 | 35 | 33655 | 3534.29 | 0.28 |
| LINE:R1 | 147 | 190776 | 24 | 5187 | 1297.8 | 0.48 |
| LINE:Penelope | 27 | 60712 | 154 | 4493 | 2248.59 | 0.15 |
| DNA:TcMar-Sagan | 71 | 50043 | 41 | 3210 | 704.83 | 0.13 |
| DNA:TcMar | 78 | 31668 | 72 | 599 | 406 | 0.08 |
| DNA:Sola | 82 | 47228 | 12 | 2521 | 575.95 | 0.12 |
| DNA:Academ | 607 | 194442 | 29 | 9078 | 320.33 | 0.49 |
| All RepeatTypes | 10707 | 5966479 | 6 | 53387 | 557.25 | 15.05 |

**Table S3.** Genetic Prediction Results.

| Feature | Feature type | Region Count | Base Count | Min Length | Max Length | Average Length | Percentage In Genome (%) |
|---|---|---|---|---|---|---|---|
| Repeat | Simple repeat | 4972 | 212698 | 6 | 321 | 42.78 | 0.54 |
| Coding | gene | 9872 | 17310604 | 3 | 38427 | 1753.51 | 43.67 |

| | | | | | | | |
|---|---|---|---|---|---|---|---|
| Genes | | | | | | | |
| Coding Genes | CDS | 70570 | 15697970 | 3 | 9625 | 222.45 | 39.61 |
| Repeat | Unknown | 2103 | 1579394 | 26 | 23688 | 751.02 | 3.98 |

**Table S4.** Gene annotation ration staistics.

| Database | Number of genes | Percentage(%) |
|---|---|---|
| Annotated in NR | 9844 | 92.58 |
| Annotated in KOG | 4633 | 43.57 |
| Annotated in CDD | 3539 | 33.28 |
| Annotated in PFAM | 4685 | 44.06 |
| Annotated in GO | 3322 | 31.24 |
| Annotated in KEGG | 2100 | 19.75 |
| Annotated in CAZy | 246 | 2.31 |
| Annotated in VFDB A | 120 | 1.13 |
| Annotated in VFDB B | 234 | 2.2 |
| Annotated in CARD | 65 | 0.61 |
| Annotated in at least one database | 9849 | 92.63 |
| Annotated in all database | 0 | 0 |
| Total genes | 10633 | 100 |

**Table S5.** BUSCO Completeness Assessment.

| Category | Number | Percentage(%) |
|---|---|---|
| Complete BUSCOs (C) | 741 | 97.76 |
| Complete and duplicated BUSCOs (D) | 19 | 2.51 |
| Complete and single-copy BUSCOs (S) | 722 | 95.25 |
| Fragmented BUSCOs (F) | 1 | 0.13 |
| Missing BUSCOs (M) | 16 | 2.11 |
| Total BUSCO groups | 758 | 100 |
